# Potential roles of voltage-gated ion channel disruption in Tuberous Sclerosis Complex

**DOI:** 10.3389/fnmol.2024.1404884

**Published:** 2024-08-26

**Authors:** Hailey X. Egido-Betancourt, Roy E. Strowd III, Kimberly F. Raab-Graham

**Affiliations:** ^1^Department of Translational Neuroscience, Wake Forest University School of Medicine, Winston-Salem, NC, United States; ^2^Department of Neurology, Wake Forest University School of Medicine, Winston-Salem, NC, United States

**Keywords:** ion channels, potassium, calcium, sodium, tuberous sclerosis complex, epilepsy

## Abstract

Tuberous Sclerosis Complex (TSC) is a lynchpin disorder, as it results in overactive mammalian target of rapamycin (mTOR) signaling, which has been implicated in a multitude of disease states. TSC is an autosomal dominant disease where 90% of affected individuals develop epilepsy. Epilepsy results from aberrant neuronal excitability that leads to recurring seizures. Under neurotypical conditions, the coordinated activity of voltage-gated ion channels keep neurons operating in an optimal range, thus providing network stability. Interestingly, loss or gain of function mutations in voltage-gated potassium, sodium, or calcium channels leads to altered excitability and seizures. To date, little is known about voltage-gated ion channel expression and function in TSC. However, data is beginning to emerge on how mTOR signaling regulates voltage-gated ion channel expression in neurons. Herein, we provide a comprehensive review of the literature describing common seizure types in patients with TSC, and suggest possible parallels between acquired epilepsies with known voltage-gated ion channel dysfunction. Furthermore, we discuss possible links toward mTOR regulation of voltage-gated ion channels expression and channel kinetics and the underlying epileptic manifestations in patients with TSC.

## Introduction

Tuberous sclerosis complex (TSC) is an autosomal dominant disease affecting roughly 1 in 6000 live births, with an estimated prevalence of 1 in 14,000 to 1 in 25,000 (Curatolo and Moavero, 2012; Kothare Sanjeev et al., 2014). Disease causing mutations in either the *TSC1* or *TSC2* gene lead to loss of protein function (O’Callaghan et al., 2004; Curatolo et al., 2008). TSC1 and TSC2 form dimers to inhibit the activity of mammalian target of rapamycin (mTOR), composed of two complexes mTORC1 and mTORC2. Loss of either TSC1 or TSC2 leads to hyperactive mTOR signaling and tuberous malformations. More than 90% of affected individuals experience seizures over the course of their lifetime (O’Callaghan et al., 2004; Kelleher and Bear, 2008; Stafstrom et al., 2017). As the etiology as TSC is well established, mechanism based treatments such as the mTORC1 inhibitor, rapamycin and other “rapalogues”, has been the focus of several clinical trials to treat TSC-related seizures (Franz et al., 2018).

TSC patients suffer from both focal and generalized epilepsy syndromes including febrile seizures, infantile spasms, focal seizures, and absence seizures (Kothare Sanjeev et al., 2014). Seizures may arise in TSC in two possible ways. Some studies speculate that seizure activity is generated by brain malformations that result from cortical tubers, which are composed of dysmorphic neurons and gliotic cells and are commonly seen in TSC patients (Stafstrom et al., 2017; Zou et al., 2017). The tubers may be surgically removed to provide temporary relief from the seizures (Bollo et al., 2008). Second, some studies suggest that hyperactive mTOR signaling itself can disrupt the excitatory/inhibitory (E/I) balance among neuronal networks (Bateup et al., 2013). Thus, in the absence of tubers, TSC patients may be susceptible to seizure-like activity and downstream neuronal damage due to hyperactive mTORC1 signaling, further disrupting mRNA translation and protein expression (Bateup et al., 2013). Cortical tuber development has been widely studied in TSC [previously reviewed in Wong (2008) and Lu et al. (2018)]; however, little is known regarding the molecular underpinnings of hyperexcitable networks downstream of mTOR signaling, that underlie epilepsy in TSC, in the absence of cortical tubers.

For decades, dysregulation of ion channels, such as voltage-gated potassium, calcium, and sodium channels, has been suggested to be the leading cause of shifts in neuronal excitability, that underlie epilepsy (Poolos and Johnston, 2012). Recently, evidence linking mTOR signaling to ion channel expression in neurons has emerged [reviewed further in Raab-Graham and Niere (2017)]. Together, these findings have led us to ask the question of whether voltage-gated ion channels are contributing to TSC-related seizures. Herein, we discuss known voltage-gated ion channels currently associated with acquired epilepsies, but not yet understood in the context of TSC. The goal of this review will be to extrapolate and expand on the current findings of voltage-gated channels implicated in other epilepsies, where aberrant mTOR signaling occurs, while surmising their role in TSC-related seizures.

## mTOR as a putative voltage sensor

**FIGURE 1:**
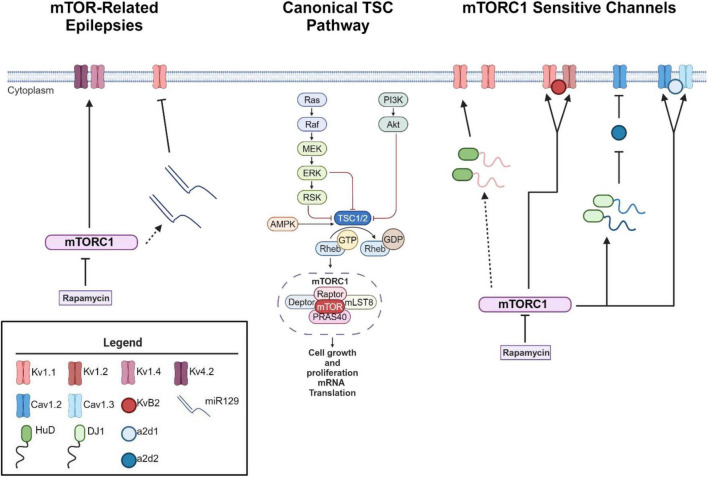
mTORC1 signaling leads to changes in ion channel expression. Schematic of canonical mTOR signaling pathway **(middle)**. mTORC1 signaling pathway represses the mRNA translation of potassium channels such that mTORC1 inhibition with the drug rapamycin increases the expression of K_v_1.1, K_v_1.2, and K_v_β2 **(right)** (Sosanya et al., 2013, 2015; Niere and Raab-Graham, 2017). Interestingly, mTOR hyperactivity differentially alters calcium influx via Ca_v_1.2, α2δ2, and Ca_v_1.3 channel expression (Hisatsune et al., 2021; Niere et al., 2023). mTOR hyperactivity leads to augmented calcium influx in the soma via an increase in Ca_v_1.3 gene and protein expression and slight increase of Ca_v_1.2 (Hisatsune et al., 2021) **(right)**. On the other hand, RNA binding protein DJ1 binds the mRNA coding for Ca_v_1.2 and α2δ2 and represses translation. This repression causes deficits in L-VGCC dendritic signaling (Niere et al., 2023). Notably, with seizure induction, blocking early-stage epileptogenesis in a model of temporal lobe epilepsy with rapamycin increases the expression of K_v_1.1 **(left)**. A secondary mechanism of repression kicks in to repress K_v_1.1 expression with extended use of rapamycin (Sosanya et al., 2015) while continued use of rapamycin restores K_v_1.4 and K_v_4.2 (Brewster et al., 2013) during late stage epileptogenesis. Dynamic expression of RNA-binding proteins and microRNAs regulate the expression of K_v_1.1. HuD increases the translation of K_v_1.1 when mTORC1 is turned off and miR-129 represses the translation of K_v_1.1 when mTORC1 is turned on. For further mechanistic detail, please see the following articles: (Sosanya et al., 2013, 2015). Interestingly, miR-129 expression increases with extended use of rapamycin, likely drives the second wave of K_v_1.1 repression and an increases in seizure frequency (Sosanya et al., 2015). Dotted lines reflect multiple molecular steps between proteins. Created with BioRender.com. Agreement number: *VB26LQZB9H.*

As mentioned above, loss of function mutations in the *TSC1* or *TSC2* genes results in hyperactive mTOR signaling (Curatolo et al., 2008; Cho, 2011; Wong, 2013; Stafstrom et al., 2017). mTOR consist of two protein complexes, mTORC1 and mTORC2. Herein, we will focus on mTORC1 signaling as it is a serine/threonine kinase that regulates mRNA translation (Curatolo et al., 2008; Curatolo and Moavero, 2012; Curatolo et al., 2015; Roach, 2016; Raab-Graham and Niere, 2017), which may alter the expression of epilepsy associated ion channels in neurons (Figure 1). mTORC1’s downstream signaling is required for many forms of synaptic plasticity, synapse formation, and recently ? Site specific expression of ion channels in neuronal dendrites (Raab-Graham et al., 2006; Cho, 2011; Brewster et al., 2013; Meng et al., 2013; Wong, 2013). Thus, an emerging theory is that mTORC1 activity may serve as a “voltage-sensor” turning on and off to maintain the membrane potential in an optimal range, through protein synthesis and repression of ion channel mRNAs (Figure 1; Niere and Raab-Graham, 2017). Thus, if mTOR activity is constitutive, as in the case of TSC, ion channel expression/repression that promotes neuronal excitability will go unchecked (Lasarge and Danzer, 2014). Epilepsy has been classically considered a disorder of ion channel dysfunction (Poolos and Johnston, 2012). Together, these data may explain why independent studies suggest that overactive mTOR signaling itself can lead to epilepsy (Zeng et al., 2008; Curatolo et al., 2015; Sosanya et al., 2015; Niere and Raab-Graham, 2017). To date, the literature is sparse in its consolidation of excessive mTOR signaling and ion channel dysfunction in TSC-related epilepsies.

## Types of TSC-associated seizures

The most common type of seizure in children with TSC are infantile spasms, occurring between 3 and 9 months after birth (Pellock et al., 2010; Appleton, 2011; Randle, 2017). Many different semiologies can be observed such as eye deviation as well as sudden bilateral and symmetrical tonic contractions, which last a few seconds (Curatolo et al., 2001). It has long been hypothesized that if these seizures are left untreated, children suffering from infantile spasms will experience impairment in developmental progress and more severe neurologic problems, such as autism spectrum disorder (Pellock et al., 2010, Alliance, 2020). Interestingly, several clinical trials have since discovered contrary findings with respect to targeting early life infantile spasms in TSC patients. One such trial found that preventative treatment with vigabatrin, the first line of treatment for infantile spasms which targets gamma-amino butyric acid (GABA)-transaminase, ultimately increases the concentration of GABA present in the brain (Yum et al., 2013). However, treatment did not delay or lower the incidence of other seizure types, such as focal and drug resistant epilepsy nor improve neurocognitive outcome at 24 months of age in TSC children (Bebin et al., 2024). On the other hand, TSC patients who showed signs of epileptiform activity before seizure onset, and were treated with vigabatrin, took longer to display clinical seizure and the preventative treatment reduced the risk of other clinical seizures (Kotulska et al., 2021); however, neurocognition was not determined. Further research targeting the underlying mechanisms of infantile spasms.

The second most common seizure type is focal onset seizures, previously called focal or partial seizures, as they originate at some specific point in the brain. These seizures differ from infantile spasms in that they can be either awareness or impaired awareness with non-motor onset or motor onset (Almobarak et al., 2018). These seizures can precede or coexist with infantile spasms, or even evolve from infantile spasms (Yum et al., 2013). While the cause of focal seizures is not fully understood, some suggest that focal insults are caused by brain malformations resulting from structural tuber alterations (Stafstrom and Carmant, 2015; Curatolo et al., 2018). To mimic focal seizures in a mouse model of TSC, pups *in utero* underwent electroporation to focally express constitutively active Rheb (Rheb*^CA^*), to augment mTOR activity only in the selected area. Indeed, this model is similar to a model of cortical dysplasia that experiences focal seizures as a result of expressing Rheb*^CA^* (Hsieh et al., 2016). The authors found that varying the concentration of Rheb led to high levels of mTOR activity, which increased seizure frequency and correlated with the degree of disease severity (Nguyen et al., 2019). These findings further the notion that focal seizures, in the absence of tuber abnormalities, is thought to be caused by select mTOR-afflicted neurons, and results in altered network excitability and seizures.

TSC patients may also suffer from generalized onset, formerly known to encompass tonic seizures, myoclonic seizures, and absence seizures (Appleton, 2011; Kiriakopoulos and Osborne, 2017). These seizures can begin focally and bilaterally expand to larger aspects of the cortex, although not necessarily the entire cortex. Patients with generalized onset seizures present with stiffened muscles, rhythmical jerking, and impaired awareness (Stafstrom and Carmant, 2015, Almobarak et al., 2018).

There is a substantial portion of TSC patients that continue to have seizures despite maximum aggressive anti-seizure treatment. For these patients, new approaches to management are needed. For example, if focal seizures coexist or precede infantile spasms, vigabatrin treatment can be less effective or have no effect. This is thought to be due to the medication only targeting one seizure type (Yum et al., 2013). Thus, it is imperative to determine the “molecular origin” of seizure onset, in order to better determine the course of treatment for a TSC affected individual.

## Clues from transcriptome studies of TSC, mTOR-mediated ion channel Expression, and speculated epilepsy

A few studies have examined the transcriptome of human cortical tubers removed from patients with TSC (Boer et al., 2010; Mills et al., 2017). Table 1 lists transcripts associated with voltage-gated channel expression (Heinemann et al., 1996; Escayg et al., 1998; Ebert et al., 2008; Zhang et al., 2015). Interestingly, all the genes listed code for auxiliary subunits that serve to increase the surface expression of the pore-forming subunit or change the ion channel kinetics. These data further convey the need to investigate the expression and function of the pore forming subunits in neuronal models of TSC. While mRNA does not necessarily mean changes in protein expression, others have demonstrated that mTOR is overactive in other models of epilepsy, similar to TSC, and these genes that code for these mTOR-dependent ion channels are summarized in Table 2 (Wang et al., 1993; McCormack et al., 1995; Burkhalter et al., 2006; Imbrici et al., 2007; Christel et al., 2012; Lee et al., 2014; Xie et al., 2014; Villa and Combi, 2016; Punetha et al., 2019; Dahimene et al., 2022). Finally, in Table 3, we propose a list of putative voltage-gated ion channels that may be dysregulated in TSC (Serôdio and Rudy, 1998; Wappl et al., 2002; Jarnot and Corbett, 2006; Ogiwara et al., 2007, 2018; Estacion et al., 2010; Smets et al., 2015; Wang et al., 2016; Wormuth et al., 2016; Fan et al., 2017; Zhang et al., 2020). Although, currently, there is no direct evidence of the potential role of dysfunction in the following voltage-gated ion channels leading to the hyperexcitable pathology in TSC, we speculate that these channels may play a role in the different seizure types present in TSC patients throughout their lives.

**TABLE 1:** Determined TSC ion channel transcripts associated with epilepsy.

Gene name	Channel type	Localization	Seizure classification	Function	Direction noted	References
*KCNAB1*	K_v_β1	Brain	Early-onset epilepsy	Inactivation regulator of alpha potassium channels	Increase	Heinemann et al., 1996; Zhang et al., 2015
*CACNB2*	Ca_v_β2	Cardiac, skeletal, smooth, and brain	Epilepsy	Modulation the gating of alpha calcium channels	Increase	Ebert et al., 2008
*CACNB4*	Ca_v_β4	Brain	Absence epilepsy; idiopathic generalized epilepsy; juvenile myoclonic epilepsy	Modulation the gating of alpha calcium channels	Increase	Burgess et al., 1997; Escayg et al., 1998

This table represents determined TSC ion channel transcripts from Boer et al. (2010) (fold change reported) and epilepsy associated genes (Wang et al., 2017) (no fold change reported).

**TABLE 2:** mTOR dependent voltage-gated ion channels associated with seizures.

Gene name	Channel type	Localization	Seizure classification	Function	Observed expression	Effect on neuronal activity	References
*KCNA1*	K_v_1.1	Brain	Epilepsy; generalized or partial	Initiation and propagation, shaping, regulating action potential	Decrease	Increase	Wang et al., 1993; Robbins and Tempel, 2012; Villa and Combi, 2016; Niere and Raab-Graham, 2017
*KCNA2*	K_v_1.2	Brain	Myoclonic epilepsy	Initiation and propagation, shaping, regulating action potential; Inactivation regulator of alpha potassium channels	Decrease	Increase	Wang et al., 1993; Robbins and Tempel, 2012; Villa and Combi, 2016; Niere and Raab-Graham, 2017
*KCNA4*	K_v_1.4	Brain	Episodic Ataxia; Epilepsy	Regulates presynaptic neurotransmitter release; regulates intrinsic excitability	Decrease	Increase	Imbrici et al., 2007; Brewster et al., 2013; Xie et al., 2014
*KCND2*	K_v_4.2	Brain	Infant-onset Epilepsy	Determine the extent of inactivation for the cell	Decrease	Increase	Burkhalter et al., 2006; Lee et al., 2014
*KCNAB2*	K_v_β2	Brain	Epilepsy	Inactivation regulator of alpha potassium channels	Decrease	Increase	McCormack et al., 1995
*CACNA1C*	Ca_v_1.2	Cardiac, smooth muscle, neuronal, adrenal, chromaffin cells	Febrile seizures	Regulates cardiac action potential; excitation-coupling	Increase (somatic); Decrease (dendritic)	Increase	Christel et al., 2012; Hisatsune et al., 2021; Niere et al., 2023
*CACNA1D*	Ca_v_1.3	Endocrine, neuronal, adrenal, chromaffin cells	Epilepsy-Associated	Sinoatrial pacemaking	Increase	Increase	Christel et al., 2012; Hisatsune et al., 2021
*CACNA2D1*	α2δ1	Skeletal muscle, brain	Epilepsy, cerebellar ataxia	Regulates VGCC current density, and activation/ inactivation kinetics	Increase	Increase	Niere et al., 2023; human protein atlas; Dahimene et al., 2022
*CACNA2D2*	α2δ2	Lung, brain	Epileptic encephalopathy, ataxia	Regulates VGCC current density, and activation/ inactivation kinetics	Decrease	Increase	Niere et al., 2023; human protein atlas; Punetha et al., 2019

This table represents mTOR modulated voltage-gated ion channels that have a potential role in TSC-associated seizure etiologies. ND, not determined.

**TABLE 3:** Voltage-gated ion channels involved in epilepsy-etiologies associated but undetermined in TSC.

Gene Name	Channel type	Localization	Seizure classification	Function	Predicted channel function	References
*KCND3*	K_v_4.3	Cardiac muscle, brain	Generalized epilepsy	Determine the extent of inactivation for the cell	GOF	Serôdio and Rudy, 1998; Smets et al., 2015
*CACNA1A*	Ca_v_2.1	Neuronal	Absence seizures	Neurotransmitter release	LOF	Wappl et al., 2002, Imbrici et al., 2004
*CACNA1E*	Ca_v_2.3	Neuronal	Absence epilepsy, human juvenile myoclonic epilepsy	Neurotransmitter release	GOF	Wormuth et al., 2016
*CACNA1G*	Ca_v_3.1	Neuronal, cardiac	Absence seizures	Sleep regulation; pacemaking	GOF	Chen et al., 2014; Wang et al., 2016
*CACNA1H*	Ca_v_3.2	Neuronal, cardiac	Absence seizures	Regulation of neuronal firing; pacemaking activity	GOF	Chen et al., 2014; Fan et al., 2017
*SCN1A*	Na_v_1.1	Brain	Myoclonic epilepsy (Dravet syndrome); generalized epilepsy with generalized clonic seizures	Generation and propagation of action potentials	LOF	Ogiwara et al., 2007
*SCN2A*	Na_v_1.2	Brain	Atypical generalized epilepsy; febrile seizures	Generation and propagation of action potentials	GOF	Jarnot and Corbett, 2006; Ogiwara et al., 2018
*SCN3A*	Na_v_1.3	Brain	Cryptogenic partial epilepsy-associated;	Generation and propagation of action potentials	GOF and LOF	Estacion et al., 2010; Menezes et al., 2020
*SCN8A*	Na_v_1.6	Brain	Infantile epilepsy	Generation and propagation of action potentials	GOF	Menezes et al., 2020
*SCN9A*	Na_v_1.7	Brain	Febrile seizures; afebrile seizures, generalized tonic-clonic seizures, myoclonic or tonic seizures, focal clonic seizures	Generation and propagation of action potentials	GOF and LOF	Yang et al., 2018; Zhang et al., 2020

GOF, gain of function; LOF, loss of function.

### Voltage-gated potassium channels

Voltage-gated potassium (K_*v*_) channels represent the largest family of genes in the K_*v*_ channel family that set the resting membrane potential and repolarize action potentials (Cooper, 2012; Robbins and Tempel, 2012). In general, K_*v*_ channels dampen neuronal activity, so loss of function (LOF) mutations lead to hyperexcitabile circuits and seizures. Potassium channels have different family subtypes that have distinct but similar function. The potassium channel consists of four α subunits and can include four cytoplasmic auxiliary β subunits (Robbins and Tempel, 2012:1). The different configurations of α and β subunits create different properties that dictate their biophysical properties including voltage-sensing and gating properties, described below.

#### K_*v*_1

*KCNA1* (K_*v*_1.1) and *KCNA2* (K_*v*_1.2) have been associated with epilepsy (Cooper, 2012; Robbins and Tempel, 2012; Boutry-Kryza et al., 2015). The Kcna family codes for the pore-forming “K_*v*_1” subunits, which may compose either A-type or delayed rectifier channels (Raab-graham and Niere, 2017). A-type currents are rapidly activating and fast inactivating, while delayed rectifiers open slowly and remain open (Raab-Graham and Niere, 2017). Depending on the brain region and cellular composition, the most abundantly expressed α subunits of the K_*v*_1 subfamily are K_*v*_1.1, K_*v*_1.2, and K_*v*_1.4 (Robbins and Tempel, 2012:1). Interestingly, K_*v*_1.1 codes for a delayed rectifier, while K_*v*_1.4 codes for the A type family. However, K_*v*_1.1 in conjunction with a K_*v*_β1 or K_*v*_1.4 subunit, can have properties of the A type family (D’Adamo et al., 2020). K_*v*_1 channels are responsible for resetting the resting membrane potential and titrating synaptic release in neurons (Foust et al., 2011; Robbins and Tempel, 2012). Interestingly, reduced expression of either K_*v*_1.1 and K_*v*_1.2 channels have been associated with epilepsy, as each knockout mouse presents with seizures that resemble the development of human epilepsy (Robbins and Tempel, 2012). Additionally, mutations in K_*v*_ channels that disrupt coassembly with other α or β subunits reduces channel functional and/or expression (D’Adamo et al., 2020). Together, Kv1 channels prevent “runaway” depolarization, increased firing rates, and excessive neurotransmitter release, all of which can lead to seizures.

It should be noted, that cross referencing the transcriptome of human TSC cortical tubers and those genes with associated epilepsies, transcripts coding for K_*v*_ auxiliary subunits K_*v*_β1 and K_*v*_β2 were detected (Tables 1, 2; Boer et al., 2010). Interestingly, other K_*v*_ subunits such as K_*v*_1.1, K_*v*_1.2, K_*v*_1.4, and K_*v*_4.2 in have been implicated in mTOR-related epilepsy models (Table 2; Brewster et al., 2013; Niere and Raab-Graham, 2017). Together, these data suggest that the K_*v*_1 class should be further investigated in TSC.

#### K_*v*_4

Among the K_*v*_4 subunits, *KCND2* (K_*v*_4.2) and *KCND3* (K_*v*_4.3) also belong to A-type voltage-gated potassium channel class. These channels are abundantly found in the nervous system within somatodendritic compartment of neurons (Zemel et al., 2018). Interestingly, several studies have shown that down regulation of the A-current leads to increased excitability (Bernard et al., 2004; Liu et al., 2014). For example, a mutation in K_*v*_4.2 (V404M) leads to impairments in inactivation after channel opening. This mutation has been associated with infant-onset epilepsy and autism (Lin et al., 2018). Thus, examining the K_*v*_4 subunit class maybe be a potential interest to TSC and associated seizure types. Altogether, we predict that these channels to be dysfunctional in TSC, specifically the potassium genes listed in Tables 1–3.

#### The role of mTOR in regulating Kv channel expression

Since the discovery of on demand local protein synthesis occurring at the synapse, dysregulation of protein synthesis can lead to misexpression of ion channel subunits and alter the membrane potential and consequently lead to seizure-like conditions (Switon et al., 2017). Pathways involving mTOR, are known to regulate local synthesis at the synapse. Under conditions where mTOR is active, local synthesis of K_*v*_1.1 is repressed on dendrites without altering axonal expression (Raab-Graham et al., 2006; Niere and Raab-Graham, 2017). Additionally, others have shown that both K_*v*_1.2, and K_*v*_β2 at the synapse are reduced when mTOR is active (Niere and Raab-Graham, 2017). Together, these findings suggest that mTOR activity toggles expression of potassium channels as a local feedback mechanism that ensures optimized synaptic function (Raab-Graham and Niere, 2017).

If mTOR activity is left unregulated, as seen in TSC, repression of K_*v*_ channel expression may lead to an increase in neuronal excitability and to eventual epileptogenesis (Cho, 2011; Meng et al., 2013; Jeong and Wong, 2016). This is suggested by Brewster and colleagues who utilized a model of pilocarpine-induced status epilepticus (SE) model and examined ion channel expression in presence and absence of rapamycin, an mTOR inhibitor. With the development of epileptogenesis, reduced expression of K_*v*_1.4 and K_*v*_4.2 in the hippocampus was observed. With the addition of rapamycin, which has been shown to reduce seizure frequency (Zeng et al., 2009) in SE rodents, protein levels of K_*v*_1.4 and K_*v*_4.2 increases to similar levels as seen in the vehicle treated rodents (Brewster et al., 2013). Altogether, these independent studies indicate that alterations in K_*v*_ expression could result in altered neuronal excitability and further studies are needed to implicate K_*v*_ channels to seizures such as those experienced in TSC.

### Voltage gated sodium channels

Voltage-gated sodium channels are responsible for the generation and the propagation of action potentials along nerve cells (Catterall, 2000; Catterall et al., 2005). Mutations in sodium channel genes most commonly augment neuronal excitability leading to epilepsy (Menezes et al., 2020). The sodium channel is a transmembrane channel consisting of an α subunit and an auxiliary β subunit (Yu and Catterall, 2003; Mantegazza and Catterall, 2012). The α subunit contains four homologous domains composed of a voltage-sensing component and a pore-forming component which undergoes modifications by the auxiliary β subunit (Catterall, 2000; Catterall et al., 2005). There are nine sodium channel isoforms; however, only the sodium channels directly implicated in excitability will be mentioned here (Table 3), and described below.

#### Na_*v*_1.1 and Na_*v*_1.2

Of interest are Na_*v*_1.1 and Na_*v*_1.2, channels expressed in neurons, but more specifically the gene mutations affecting the α subunits of these channels. These mutations lead to inherited forms of epilepsy that differ based on type of α subunit defect (Mantegazza and Catterall, 2012). The *SCN1A* gene, which encodes the Na_*v*_1.1 channel, has been associated with Dravet syndrome, which displays afebrile intractable seizures (Craig et al., 2012; Schmunk and Gargus, 2013). Missense mutations in the *SCN1A* gene (D322N), commonly display gain of function (GOF), that lead to enhanced sodium currents as a result of lack of inhibition on excitatory neurons (Menezes et al., 2020). Likewise, mutations in the *SCN2A* gene (A467T), that encodes the voltage-gated sodium channel Na_*v*_1.2, have been shown to elicit seizure behavior such as in generalized epilepsy with febrile seizure plus (GEFS+) syndrome by also enhancing sodium currents (Schmunk and Gargus, 2013; Wolff et al., 2017). Additionally, LOF mutations in *SCN2A* have been linked to ASD and intellectual disability, all of which are commonly seen in patients with TSC. Thus, further research is needed to understand the mechanisms by which mutations in these genes leads to TSC.

#### Na_*v*_1.3, Na_*v*_1.6, and Na_*v*_1.7

Other voltage-gated sodium channels, such as Na_*v*_1.6 and Na_*v*_1.7 are associated with infantile spasms and febrile seizures, respectively, while Na_*v*_1.3 has been associated with patients with epilepsy (Menezes et al., 2020). Notably, mutations in *SCN8A* gene, coding for Na_*v*_1.6, affects the action potential threshold which increases spontaneous and repetitive firing leading to an increase in excitability (Menezes et al., 2020). Additionally, GOF and LOF mutations in Na_*v*_1.3 and Na_*v*_1.7, have been reported to alter the biophysical properties of neurons as these genes modify other sodium channels such as Na_*v*_1.1, which can contribute to pathogenesis of epilepsy, whoever, more studies are needed to ascertain their direct involvement. Altogether, these channels are associated with types of seizures experienced within TSC, however, these channels remain uninvestigated, making these channels possible candidates to examine in TSC.

### Voltage-gated calcium ion channels

#### Speculated voltage-gated ion channels in TSC associated epilepsy

Voltage-gated calcium channels are required for different functions in the neuron, such as controlling neuronal excitability and regulating calcium-sensitive intracellular pathways (Cain and Snutch, 2012; Zamponi et al., 2015). There are three classes of voltage-gated calcium channels, Ca_*v*_1, Ca_*v*_2, and Ca_*v*_3 (Table 3). Each class has subclasses of ion channel expression that vary based function, and kinetics. The channels are either high voltage (HVA) or low voltage activated (LVA), meaning the channel opens or activates at −40 and −60 mV, respectively (Cain and Snutch, 2012). The calcium channel, like the sodium and potassium channel, contain an α subunit that stands as the pore-forming unit that is selective for calcium (Cain and Snutch, 2012; Zamponi et al., 2015). They also have auxiliary subunits β, γ and α2δ that regulate the properties of the channel (Campiglio and Flucher, 2015). Because of the genetic diversity among the calcium channels, only a select few of the channels expressed in the brain will be discussed, specifically the HVA and LVA channel subunits listed in Tables 1–3.

The β auxiliary subunits play an important role in enhancing the biophysical properties of the α subunit, such as channel folding, channel trafficking, and alters gating kinetics and voltage-dependence (Dolphin, 2003, 2016). Interestingly, our comparison of the human TSC cortical tuber transcriptome cross referenced with genes associated with epilepsies, yielded elevated mRNA coding for Ca_*v*_β2 and Ca_*v*_β4 (Table 1; Boer et al., 2010). Interestingly, one study demonstrated that ablation of Ca_*v*_β1, Ca_*v*_β2, and Ca_*v*_β3 have no major impact on neuronal function (Ball et al., 2002; Vergnol et al., 2022). On the other hand, one study demonstrated Ca_*v*_β4 is associated with the *lethargic* mouse model of epilepsy (Burgess et al., 1997; Vergnol et al., 2022), while another study showed that disruption in the Ca_*v*_β2 gene leads to diminished L-type channel currents (Weissgerber et al., 2006). Although the biophysical properties of these two subunits have yet to be determined in TSC, this finding shows possible insights into voltage-gated calcium channels and whether they are disrupted.

#### Ca_*v*_2

The Cav2 family encompasses Ca_*v*_2.1, Ca_*v*_2.2, and Ca_*v*_2.3 isoforms. these channels are comprised of a pore-forming α subunit and auxiliary β subunits. Together, they are responsible for regulating Ca2+ entry in response to depolarization and release of neurotransmitters (Mochida, 2019). These channels can undergo alternative splicing, and thus, have a wide spectrum of biophysical properties. Of particular interest are Ca_*v*_2.1 and Ca_*v*_2.3, as shown in Table 3, will be discussed further as these channels have more direct implications to seizure. There are several Ca_*v*_2.1 channel mutations that generate epileptic phenotypes commonly seen within TSC. For example, TSC patients can suffer from absence epilepsy, whose mouse models, “leaner”, “tottering”, and “rocker,” display epileptic phenotypes as a result of different Ca_*v*_2.1 channel mutations (Imbrici et al., 2004; Mochida, 2019). These mutations affect Ca_*v*_2.1 current density by slowing channel inactivation as well as imbalances on inhibitory to excitatory neurotransmission leading to increased firing (Rajakulendran and Hanna, 2016). Similarly, Ca_*v*_2.3 has also been demonstrated to play a role in absence epilepsy role (Zaman et al., 2011). Nevertheless, the contribution of Ca_*v*_2.1 or Ca_*v*_2.3 to TSC absence epilepsy remains to be determined and, thus, this remains a possible avenue of exploration.

#### Ca_*v*_3

T-type calcium channels, do not require auxiliary subunits (Simms and Zamponi, 2014). Because the Ca_*v*_3 subunits have been shown to undergo alternative splicing, resulting in channel function diversity, the T-type channels that will be discussed in the context of TSC will be Ca_*v*_3.1 (*CACNA1G)* and Ca_*v*_3.2 (*CACNA1H)*. *CACNA1G* channels are highly expressed in thalamocortical (TC) neurons (Chen et al., 2014). *CACNA1H* has been shown to be primarily expressed in the dorsal root ganglion, dentate of the hippocampus, and thalamus (Graef et al., 2011; Simms and Zamponi, 2014; Bernal Sierra et al., 2017). Ca_*v*_3.2 mutations have been shown to lead to seizures in murine models, specifically absence epilepsy. Because of this implication in seizure commonality, and because this limbic seizure can precede from subcortical structures such as the thalamus, it is possible that T-channels play a role in initiating the spread to higher structures in the TSC brain.

#### Ca_*V*_ channel expression in TSC

**FIGURE 2:**
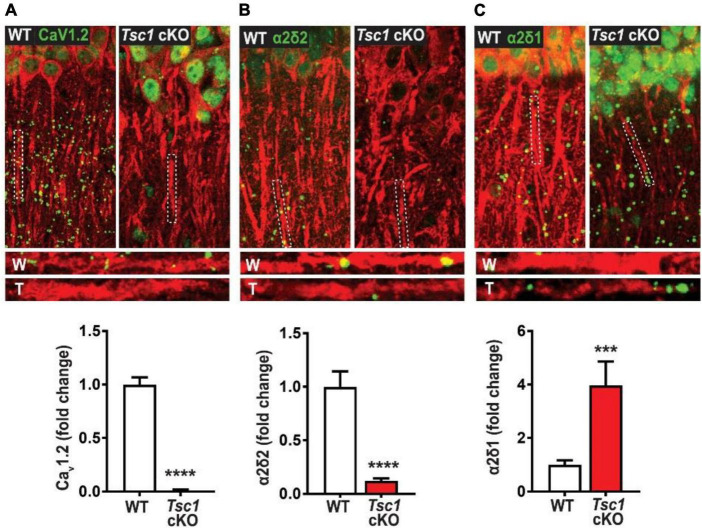
Tsc1 cKO mouse model exhibits decreased *de novo* protein synthesis of Ca_V_1.2 *α2δ2, but not α2δ1* (40). De novo protein synthesis, visualized by Surface Sensing of Translation-Proximity Ligation Assay (SUnSET-PLA) (green) in hippocampal dendrites (MAP2, red). The SUnSET-PLA combinatory assay labels newly synthesized Ca_V_1.2, α2δ2, and α2δ1 proteins in the hippocampus by detecting puromycin (which binds and halts translation) on a translating ribosome and the translating protein with a specific antibody. This assay allows one to separate new protein from already synthesize protein. WT (W) and TSC (T) dendrites are outlined by broken lines. **(A)** Basal Ca_V_1.2 protein synthesis is detected in dendrites of WT is markedly reduced in TSC. **(B)** α2δ2 basal new protein synthesis is detected in dendrites of WT but is attenuated in TSC. **(C)** Basal α2δ1 protein synthesis in dendrites of WT is lower than TSC. For representative images in panel **(A)** through panel **(C)**, Ca_V_1.2, α2δ2, and α2δ1 puncta were dilated once using ImageJ. Adapted from Niere et al. (2023). Bar values represent mean ± SEM. ***P < 0.001, ****P < 0.0001.

As previously mentioned above, the HVA class also encompasses L-type calcium channels as they are integral to cell’s membrane complex that mediate influx of Ca2+ after a depolarization response (Hofmann et al., 2014). The “L” in L-type represents the long-lasting inward currents during depolarization which have distinguished them from their “transient current” T-type cousins (Zamponi et al., 2015). One L-type channel, Ca_*v*_1.2, is composed of three subunits α1, α2δ, and β axillary subunits (Hofmann et al., 2014; Zamponi et al., 2015), and may provide more insight into TSC epileptic phenotypes. There have been case studies indicating the presence of febrile seizures among TSC patients (Kubo et al., 2011; Siddaraju et al., 2016). However, these case studies primarily served as documentation for the patients’ condition, and no other studies have followed up on the possibilities of febrile seizures in TSC models. Interestingly, one independent study demonstrated that febrile seizures in rat pups may be prevented with the use of nimodipine (Radzicki et al., 2013). Additionally, mTOR hyperactivation has been shown to differentially regulate L channel expression in TSC. An independent study has demonstrated that somatic Ca_*v*_1.2 and Ca_*v*_1.3 gene and protein expression are augmented in TSC2-null neurons. Hisatsune et al., 2021 also demonstrates that Cav1.3 triggers enhanced neuronal activity of TSC2^–/–^ neurons and could be a potential novel target for epilepsy in TSC (Hisatsune et al., 2021). On the other hand, Ca_*v*_1.2 *de novo* protein synthesis was found to be reduced in the dendrites of hippocampal CA-1 neurons in a mouse model of TSC1 (Figure 2). Furthermore, Niere et al. (2023) found that an RNA binding protein DJ-1 coordinates the expression of Ca_*v*_ channel complex, including Cav1.2 and α2δ2, resulting in attenuated calcium signaling in the dendrites (Figure 2 and Table 2). Like the β subunits mentioned above, the α2δ auxiliary subunits play an important role in trafficking and gating of the α subunits (Dolphin, 2003, 2016). Additionally, α2δ1 was found to be overexpressed in conditionally knockout TSC hippocampal dendrites (Niere et al., 2023); however its role in TSC has not been established. Together, these two studies on L-type calcium channels, suggest that subcellular localization of these channels differentially affects calcium influx across the cell. Considering the importance of calcium channel to seizures, these findings give credence to further investigation of calcium channel dysfunction in TSC.

## Conclusion

In conclusion, the disruption of voltage-gated ion channels leads to different types of seizures. mTOR, downstream of the TSC, has been shown to be involved in regulating ion channel expression, and may contribute to epileptogenesis. Because of the complexity each voltage-gated ion channel, there are many unanswered questions of their role in TSC. Yet, understanding the contribution from each voltage-gated ion channel may provide insight into the heterogeneity of seizures in TSC and possibly determine new therapeutic targets of interest.

## Scope statement

Tuberous sclerosis complex (TSC) is a neurodevelopmental disorder that results in hyperactive mammalian/mechanistic target of rapamycin (mTOR) signaling leading to altered neuronal excitability and seizures; however, the underlying mechanisms remain a mystery. Several potassium, sodium, or calcium voltage-gated channels have been found to be causative in disorders that result in aberrant neuronal excitability and seizures. Surprisingly, these channels remain understudied in TSC-associated neuronal dysfunction. The coordination of these ionic conductances, dictated by the channel’s expression, subcellular localization, and biophysical properties, keep neurons operating in an optimal range, providing network stability. Our review examines the current TSC literature describing common seizure types, clinical trials, and genomic studies that potentially implicate potassium, sodium, and calcium voltage-gated channel dysfunction in TSC. Notably, the expression of several voltage-gated ion channels and auxiliary subunits have been shown to be regulated by mTOR signaling, arguing for further studies of ion channel dysfunction in TSC. *Frontiers in Molecular Neuroscience-Molecular Signalling and Pathways* is particularly interested in topics that pertain to brain disease mechanisms such as TSC, molecular signaling pathways such as mTOR, and synaptic and cellular proteins such as voltage-gated ion channels.

## Data availability statement

The original contributions presented in this study are included in the article/supplementary material, further inquiries can be directed to the corresponding author.

## Author contributions

HE-B: Conceptualization, Data curation, Writing – original draft, Writing – review and editing. RS: Writing – review and editing. KR-G: Conceptualization, Funding acquisition, Supervision, Writing – original draft, Writing – review and editing.

## References

[B1] Alliance T. S. (2020). *Infantile spasms [Online].* Available online at: https://www.tsalliance.org/about-tsc/signs-and-symptoms-of-tsc/brain-and-neurological-function/infantile-spasms/ (accessed October 06, 2020).

[B2] Almobarak S. Almuhaizea M. Abukhaled M. Alyamani S. Dabbagh O. Chedrawi A. (2018). Tuberous sclerosis complex: Clinical spectrum and epilepsy: A retrospective chart review study. *Transl. Neurosci.* 9 154–160. 30479846 10.1515/tnsci-2018-0023 PMC6234476

[B3] Appleton R. E. (2011). *Tsc and epilepsy [Online].* Available online at: https://tuberous-sclerosis.org/wp-content/uploads/2019/10/TSA-TSC-and-epilepsy.pdf (accessed October 6, 2020).

[B4] Ball S. L. Powers P. A. Shin H. S. Morgans C. W. Peachey N. S. Gregg R. G. (2002). Role of the beta(2) subunit of voltage-dependent calcium channels in the retinal outer plexiform layer. *Invest. Ophthalmol. Vis. Sci.* 43 1595–1603. 11980879

[B5] Bateup H. S. Johnson C. A. Denefrio C. L. Saulnier J. L. Kornacker K. Sabatini B. L. (2013). Excitatory/inhibitory synaptic imbalance leads to hippocampal hyperexcitability in mouse models of tuberous sclerosis. *Neuron* 78 510–522. 10.1016/j.neuron.2013.03.017 23664616 PMC3690324

[B6] Bebin E. M. Peters J. M. Porter B. E. Mcpherson T. O. O’kelley S. Sahin M. (2024). Early treatment with vigabatrin does not decrease focal seizures or improve cognition in tuberous sclerosis complex: The prevent trial. *Ann. Neurol.* 95, 15–26. 10.1002/ana.26778 PMC10899525 37638552

[B7] Bernal Sierra Y. A. Haseleu J. Kozlenkov A. Bégay V. Lewin G. R. (2017). Genetic Tracing of Ca(v)3.2 T-type calcium channel expression in the peripheral nervous system. *Front. Mol. Neurosci.* 10:70. 10.3389/fnmol.2017.00070 28360836 PMC5350092

[B8] Bernard C. Anderson A. Becker A. Poolos N. P. Beck H. Johnston D. (2004). Acquired dendritic channelopathy in temporal lobe epilepsy. *Science* 305 532–535. 15273397 10.1126/science.1097065

[B9] Boer K. Crino P. B. Gorter J. A. Nellist M. Jansen F. E. Spliet W. G. M. (2010). Gene expression analysis of tuberous sclerosis complex cortical tubers reveals increased expression of adhesion and inflammatory factors. *Brain Pathol.* 20 704–719. 19912235 10.1111/j.1750-3639.2009.00341.x PMC2888867

[B10] Bollo R. J. Kalhorn S. P. Carlson C. Haegeli V. Devinsky O. Weiner H. L. (2008). Epilepsy surgery and tuberous sclerosis complex: Special considerations. *Neurosurg. Focus* 25:E13. 10.3171/FOC/2008/25/9/E13 18759614

[B11] Boutry-Kryza N. Labalme A. Ville D. De Bellescize J. Touraine R. Prieur F. (2015). Molecular characterization of a cohort of 73 patients with infantile spasms syndrome. *Eur. J. Med. Genet.* 58 51–58. 10.1016/j.ejmg.2014.11.007 25497044

[B12] Brewster A. L. Lugo J. N. Patil V. V. Lee W. L. Qian Y. Vanegas F. (2013). Rapamycin reverses status epilepticus-induced memory deficits and dendritic damage. *PLoS One* 8:e57808. 10.1371/journal.pone.0057808 23536771 PMC3594232

[B13] Burgess D. L. Jones J. M. Meisler M. H. Noebels J. L. (1997). Mutation of the Ca2+ channel β subunit gene cchb4 is associated with ataxia and seizures in the lethargic (lh) mouse. *Cell* 88 385–392. 9039265 10.1016/s0092-8674(00)81877-2

[B14] Burkhalter A. Gonchar Y. Mellor R. L. Nerbonne J. M. (2006). Differential expression of I(A) channel subunits Kv4.2 and Kv4.3 in mouse visual cortical neurons and synapses. *J. Neurosci.* 26 12274–12282. 10.1523/JNEUROSCI.2599-06.2006 17122053 PMC6675432

[B15] Cain S. M. Snutch T. P. (2012). *Voltage-gated calcium channels in epilepsy. Jasper’s basic mechanisms of the epilepsies*, 4th Edn. Bethesda, MD: National Center for Biotechnology Information (US). 22787663

[B16] Campiglio M. Flucher B. E. (2015). The Role of Auxiliary Subunits for the Functional Diversity of Voltage-Gated Calcium Channels. *J. Cell. Physiol.* 230 2019–2031. 25820299 10.1002/jcp.24998 PMC4672716

[B17] Catterall W. A. (2000). From ionic currents to molecular mechanisms. *Neuron* 26 13–25. 10798388 10.1016/s0896-6273(00)81133-2

[B18] Catterall W. A. Goldin A. L. Waxman S. G. (2005). International union of pharmacology. XLVII. Nomenclature and structure-function relationships of voltage-gated sodium channels. *Pharmacol. Rev.* 57:397. 10.1124/pr.57.4.4 16382098

[B19] Chen Y. Parker W. D. Wang K. (2014). The role of T-type calcium channel genes in absence seizures. *Front. Neurol.* 5 45–45. 10.3389/fneur.2014.00045 24847307 PMC4023043

[B20] Cho C.-H. (2011). Frontier of epilepsy research - mtor signaling pathway. *Exp. Mol. Med.* 43 231–274. 21467839 10.3858/emm.2011.43.5.032 PMC3104248

[B21] Christel C. J. Cardona N. Mesirca P. Herrmann S. Hofmann F. Striessnig J. (2012). Distinct localization and modulation of Cav1.2 and Cav1.3 L-type Ca2+ channels in mouse sinoatrial node. *J. Physiol.* 590 6327–6342. 10.1113/jphysiol.2012.239954 23045342 PMC3533195

[B22] Cooper E. C. (2012). *Potassium channels (including Kcnq) and epilepsy. Jasper’s basic mechanisms of the epilepsies*, 4th Edn. Bethesda, MD: National Center for Biotechnology Information (US). 22787644

[B23] Craig A. K. De Menezes M. S. Saneto R. P. (2012). Dravet syndrome: Patients with co-morbid Scn1A gene mutations and mitochondrial electron transport chain defects. *Seizure Eur. J. Epilepsy* 21 17–20. 10.1016/j.seizure.2011.08.010 21906962

[B24] Curatolo P. Moavero R. (2012). mTOR inhibitors in tuberous sclerosis complex. *Curr. Neuropharmacol.* 10 404–415. 23730262 10.2174/157015912804143595 PMC3520048

[B25] Curatolo P. Bombardieri R. Jozwiak S. (2008). Tuberous sclerosis. *Lancet* 372 657–668. 18722871 10.1016/S0140-6736(08)61279-9

[B26] Curatolo P. Moavero R. De Vries P. J. (2015). Neurological and neuropsychiatric aspects of tuberous sclerosis complex. *Lancet Neurol.* 14 733–745. 26067126 10.1016/S1474-4422(15)00069-1

[B27] Curatolo P. Moavero R. Van Scheppingen J. Aronica E. (2018). mTOR dysregulation and tuberous sclerosis-related epilepsy. *Expert rev. Neurother.* 18:185. 10.1080/14737175.2018.1428562 29338461

[B28] Curatolo P. Seri S. Verdecchia M. Bombardieri R. (2001). Infantile spasms in tuberous sclerosis complex. *Brain Dev.* 23 502–507. 11701245 10.1016/s0387-7604(01)00300-x

[B29] D’Adamo M. C. Liantonio A. Rolland J. F. Pessia M. Imbrici P. (2020). Kv1.1 channelopathies: Pathophysiological mechanisms and therapeutic approaches. *Int. J. Mol. Sci.* 21:2935. 10.3390/ijms21082935 32331416 PMC7215777

[B30] Dahimene S. Von Elsner L. Holling T. Mattas L. S. Pickard J. Lessel D. (2022). Biallelic Cacna2D1 loss-of-function variants cause early-onset developmental epileptic encephalopathy. *Brain* 145 2721–2729. 10.1093/brain/awac081 35293990 PMC9420018

[B31] Dolphin A. C. (2003). Subunits of voltage-gated calcium channels. *J. Bioenerget. Biomembr.* 35 599–620. 10.1023/b:jobb.0000008026.37790.5a 15000522

[B32] Dolphin A. C. (2016). Voltage-gated calcium channels and their auxiliary subunits: Physiology and pathophysiology and pharmacology. *J. Physiol.* 594 5369–5390. 27273705 10.1113/JP272262 PMC5043047

[B33] Ebert A. M. Mcanelly C. A. Srinivasan A. Mueller R. L. Garrity D. B. Garrity D. M. (2008). The calcium channel β2 (Cacnb2) subunit repertoire in teleosts. *BMC Mol. Biol.* 9:38. 10.1186/1471-2199-9-38 18419826 PMC2365960

[B34] Escayg A. Jones J. M. Kearney J. A. Hitchcock P. F. Meisler M. H. (1998). Calcium channel β4 (CACNB4): Human ortholog of the mouse epilepsy genelethargic. *Genomics* 50 14–22. 9628818 10.1006/geno.1998.5311

[B35] Estacion M. Gasser A. Dib-Hajj S. D. Waxman S. G. (2010). A sodium channel mutation linked to epilepsy increases ramp and persistent current of Nav1.3 and induces hyperexcitability in hippocampal neurons. *Exp. Neurol.* 224 362–368. 10.1016/j.expneurol.2010.04.012 20420834

[B36] Fan J. Gandini M. A. Zhang F.-X. Chen L. Souza I. A. Zamponi G. W. (2017). Down-regulation of T-type Cav3.2 channels by hyperpolarization-activated cyclic nucleotide-gated channel 1 (HCN1): Evidence of a signaling complex. *Channels* 11 434–443. 10.1080/19336950.2017.1326233 28467171 PMC5626362

[B37] Foust A. J. Yu Y. Popovic M. Zecevic D. Mccormick D. A. (2011). Somatic membrane potential and Kv1 channels control spike repolarization in cortical axon collaterals and presynaptic boutons. *J. Neurosci.* 31 15490–15498. 10.1523/JNEUROSCI.2752-11.2011 22031895 PMC3225031

[B38] Franz D. N. Lawson J. A. Yapici Z. Brandt C. Kohrman M. H. Wong M. (2018). Everolimus dosing recommendations for tuberous sclerosis complex-associated refractory seizures. *Epilepsia* 59 1188–1197. 10.1111/epi.14085 29727013 PMC6033043

[B39] Graef J. D. Huitt T. W. Nordskog B. K. Hammarback J. H. Godwin D. W. (2011). Disrupted thalamic T-type Ca2+ channel expression and function during ethanol exposure and withdrawal. *J. Neurophysiol.* 105 528–540. 10.1152/jn.00424.2010 21148095 PMC3059161

[B40] Heinemann S. H. Rettig J. Graack H. R. Pongs O. (1996). Functional characterization of Kv channel beta-subunits from rat brain. *J. Physiol.* 493 625–633. 8799886 10.1113/jphysiol.1996.sp021409 PMC1159012

[B41] Hisatsune C. Shimada T. Miyamoto A. Lee A. Yamagata K. (2021). Tuberous sclerosis complex (TSC) inactivation increases neuronal network activity by enhancing Ca2+ Influx via L-Type Ca2+ channels. *J. Neurosci.* 41 8134–8149. 10.1523/JNEUROSCI.1930-20.2021 34417327 PMC8482857

[B42] Hofmann F. Flockerzi V. Kahl S. Wegener J. W. (2014). L-Type CaV1.2 calcium channels: From in vitro findings to in vivo function. *Physiol. Rev.* 94 303–326. 24382889 10.1152/physrev.00016.2013

[B43] Hsieh L. S. Wen J. H. Claycomb K. Huang Y. Harrsch F. A. Naegele J. R. (2016). Convulsive seizures from experimental focal cortical dysplasia occur independently of cell misplacement. *Nat. Commun.* 7:11753. 10.1038/ncomms11753 27249187 PMC4895394

[B44] Imbrici P. D’adamo M. C. Cusimano A. Pessia M. (2007). Episodic ataxia type 1 mutation F184C alters Zn2+-induced modulation of the human K+ channel Kv1.4-Kv1.1/Kvbeta1.1. *Am. J. Physiol. Cell Physiol.* 292 C778–C787. 10.1152/ajpcell.00259.2006 16956965

[B45] Imbrici P. Jaffe S. L. Eunson L. H. Davies N. P. Herd C. Robertson R. (2004). Dysfunction of the brain calcium channel CaV2.1 in absence epilepsy and episodic ataxia. *Brain* 127 2682–2692. 15483044 10.1093/brain/awh301

[B46] Jarnot M. Corbett A. M. (2006). Immunolocalization of NaV1.2 channel subtypes in rat and cat brain and spinal cord with high affinity antibodies. *Brain Res.* 1107 1–12. 10.1016/j.brainres.2006.05.090 16815341

[B47] Jeong A. Wong M. (2016). Tuberous sclerosis complex as a model disease for developing new therapeutics for epilepsy. *Expert Rev. Neurother.* 16 437–447. 10.1586/14737175.2016.1151788 26854692

[B48] Kelleher R. J. Bear M. F. (2008). The autistic neuron: Troubled translation? *Cell* 135 401–406. 10.1016/j.cell.2008.10.017 18984149

[B49] Kiriakopoulos E. S. Osborne S. (2017). *Types of seizures [Online]. Epilepsy Foundation.* Available online at: https://www.epilepsy.com/learn/types-seizures (accessed June 6, 2020).

[B50] Kothare Sanjeev V. Singh K. Chalifoux Jason R. Staley Brigid A. Weiner Howard L. Menzer K. (2014). Severity of manifestations in tuberous sclerosis complex in relation to genotype. *Epilepsia* 55 1025–1029. 24917535 10.1111/epi.12680

[B51] Kotulska K. Kwiatkowski D. J. Curatolo P. Weschke B. Riney K. Jansen F. (2021). Prevention of epilepsy in infants with tuberous sclerosis complex in the EPISTOP trial. *Ann. Neurol.* 89 304–314. 33180985 10.1002/ana.25956 PMC7898885

[B52] Kubo M. Iwashita K. Oyachi N. Oyama T. Yamamoto T. (2011). Two different types of infantile renal cell carcinomas associated with tuberous sclerosis. *J. Pediatr. Surg.* 46 e37–e41. 10.1016/j.jpedsurg.2011.06.035 22008361

[B53] Lasarge C. L. Danzer S. C. (2014). Mechanisms regulating neuronal excitability and seizure development following mtor pathway hyperactivation. *Front. Mol. Neurosci.* 7:18. 10.3389/fnmol.2014.00018 24672426 PMC3953715

[B54] Lee H. Lin M. C. Kornblum H. I. Papazian D. M. Nelson S. F. (2014). Exome sequencing identifies de novo gain of function missense mutation in Kcnd2 in identical twins with autism and seizures that slows potassium channel inactivation. *Hum. Mol. Genet.* 23 3481–3489. 10.1093/hmg/ddu056 24501278 PMC4049306

[B55] Lin M. A. Cannon S. C. Papazian D. M. (2018). Kv4.2 autism and epilepsy mutation enhances inactivation of closed channels but impairs access to inactivated state after opening. *Proc. Natl. Acad. Sci. U.S.A.* 115 E3559–E3568. 10.1073/pnas.1717082115 29581270 PMC5899440

[B56] Liu Y. Q. Huang W. X. Sanchez R. M. Min J. W. Hu J. J. He X. H. (2014). Regulation of Kv4.2 A-type potassium channels in HEK-293 cells by hypoxia. *Front. Cell. Neurosci.* 8:329. 10.3389/fncel.2014.00329 25352783 PMC4196569

[B57] Lu D. S. Karas P. J. Krueger D. A. Weiner H. L. (2018). Central nervous system manifestations of tuberous sclerosis complex. *Am. J. Med. Genet. C Semin. Med. Genet.* 178 291–298. 30230171 10.1002/ajmg.c.31647

[B58] Mantegazza M. Catterall W. A. (2012). *Voltage-gated Na+ channels: Structure, function, and pathophysiology*, 4th Edn. Bethesda, MD: National Center for Biotechnology Information (US). 22787615

[B59] McCormack K. Mccormack T. Tanouye M. Rudy B. Stühmer W. (1995). Alternative splicing of the human Shaker K+ channel beta 1 gene and functional expression of the beta 2 gene product. *FEBS Lett.* 370 32–36. 10.1016/0014-5793(95)00785-8 7649300

[B60] Menezes L. F. S. Sabiá Júnior E. F. Tibery D. V. Carneiro L. D. A. Schwartz E. F. (2020). Epilepsy-related voltage-gated sodium channelopathies: A review. *Front. Pharmacol.* 11:1276. 10.3389/fphar.2020.01276 33013363 PMC7461817

[B61] Meng X.-F. Yu J.-T. Song J.-H. Chi S. Tan L. (2013). Role of the mTOR signaling pathway in epilepsy. *J. Neurol. Sci.* 332 4–15. 23773767 10.1016/j.jns.2013.05.029

[B62] Mills J. D. Iyer A. M. Van Scheppingen J. Bongaarts A. Anink J. J. Janssen B. (2017). Coding and small non-coding transcriptional landscape of tuberous sclerosis complex cortical tubers: Implications for pathophysiology and treatment. *Sci. Rep.* 7:8089. 10.1038/s41598-017-06145-8 PMC5556011 28808237

[B63] Mochida S. (2019). Presynaptic calcium channels. *Int. J. Mol. Sci.* 20:2217. 10.3390/ijms20092217 PMC6539076 31064106

[B64] Nguyen L. H. Mahadeo T. Bordey A. (2019). mTOR hyperactivity levels influence the severity of epilepsy and associated neuropathology in an experimental model of tuberous sclerosis complex and focal cortical dysplasia. *J. Neurosci.* 39 2762–2773. 10.1523/JNEUROSCI.2260-18.2019 30700531 PMC6445990

[B65] Niere F. Raab-Graham K. F. (2017). mTORc1 is a local, postsynaptic voltage sensor regulated by positive and negative feedback pathways. *Front. Cell. Neurosci.* 11:152. 10.3389/fncel.2017.00152 28611595 PMC5447718

[B66] Niere F. Uneri A. Mcardle C. J. Deng Z. Egido-Betancourt H. X. Cacheaux L. P. (2023). Aberrant DJ-1 expression underlies L-type calcium channel hypoactivity in dendrites in tuberous sclerosis complex and Alzheimer’s disease. *Proc. Natl. Acad. Sci. U.S.A.* 120:e2301534120. 10.1073/pnas.2301534120 37903257 PMC10636362

[B67] O’Callaghan F. Harris T. Joinson C. Bolton P. Noakes M. Presdee D. (2004). The relation of infantile spasms, tubers, and intelligence in tuberous sclerosis complex. *Arch. Dis. Child.* 89 530–533. 15155396 10.1136/adc.2003.026815 PMC1719953

[B68] Ogiwara I. Miyamoto H. Morita N. Atapour N. Mazaki E. Inoue I. (2007). Nav1.1 localizes to axons of parvalbumin-positive inhibitory interneurons: A circuit basis for epileptic seizures in mice carrying an Scn1a gene mutation. *J. Neurosci.* 27:5903. 10.1523/JNEUROSCI.5270-06.2007 17537961 PMC6672241

[B69] Ogiwara I. Miyamoto H. Tatsukawa T. Yamagata T. Nakayama T. Atapour N. (2018). Nav1.2 haplodeficiency in excitatory neurons causes absence-like seizures in mice. *Commun. Biol.* 1:96. 10.1038/s42003-018-0099-2 30175250 PMC6115194

[B70] Pellock J. M. Hrachovy R. Shinnar S. Baram T. Z. Bettis D. Dlugos D. J. (2010). Infantile spasms: A U.S. consensus report. *Epilepsia* 51 2175–2189. 20608959 10.1111/j.1528-1167.2010.02657.x

[B71] Poolos N. P. Johnston D. (2012). Dendritic ion channelopathy in acquired epilepsy. *Epilepsia* 53 32–40. 23216577 10.1111/epi.12033 PMC3531827

[B72] Punetha J. Karaca E. Gezdirici A. Lamont R. E. Pehlivan D. Marafi D. (2019). Biallelic Cacna2D2 variants in epileptic encephalopathy and cerebellar atrophy. *Ann. Clin. Transl. Neurol.* 6 1395–1406. 10.1002/acn3.50824 31402629 PMC6689679

[B73] Raab-Graham K. F. Niere F. (2017). mTOR referees memory and disease through mRNA repression and competition. *FEBS Lett.* 591 1540–1554. 10.1002/1873-3468.12675 28493559 PMC5933947

[B74] Raab-Graham K. F. Haddick P. C. G. Jan Y. N. Jan L. Y. (2006). Activity- and mTOR-dependent suppression of Kv1.1 channel mRNA translation in dendrites. *Science* 314:144. 10.1126/science.1131693 17023663

[B75] Radzicki D. Yau H.-J. Pollema-Mays S. L. Mlsna L. Cho K. Koh S. (2013). Temperature-sensitive Cav1.2 calcium channels support intrinsic firing of pyramidal neurons and provide a target for the treatment of febrile seizures. *J. Neurosci.* 33 9920–9931. 10.1523/JNEUROSCI.5482-12.2013 23761887 PMC3682377

[B76] Rajakulendran S. Hanna M. G. (2016). The role of calcium channels in epilepsy. *Cold Spring Harb. Perspect. Med.* 6:a022723. 10.1101/cshperspect.a022723 PMC4691803 26729757

[B77] Randle S. C. (2017). Tuberous sclerosis complex: A review. *Pediatr. Ann.* 46 e166–e171. 28414398 10.3928/19382359-20170320-01

[B78] Roach E. S. (2016). Applying the lessons of tuberous sclerosis: The 2015 Hower award lecture. *Pediatr. Neurol.* 63 6–22. 10.1016/j.pediatrneurol.2016.07.003 27543366

[B79] Robbins C. A. Tempel B. L. (2012). Kv1.1 and Kv1.2: Similar channels, different seizure models. *Epilepsia* 53 134–141. 10.1111/j.1528-1167.2012.03484.x 22612818

[B80] Schmunk G. Gargus J. J. (2013). Channelopathy pathogenesis in autism spectrum disorders. *Front. Genet.* 4:222. 10.3389/fgene.2013.00222 24204377 PMC3817418

[B81] Serôdio P. Rudy B. (1998). Differential expression of Kv4 K+ channel subunits mediating subthreshold transient K+ (A-Type) currents in rat brain. *J. Neurophysiol.* 79 1081–1091. 9463463 10.1152/jn.1998.79.2.1081

[B82] Siddaraju M. L. S. Reddy T. Bandari A. K. (2016). A case of tuberous sclerosis - presenting as febrile seizures with status epilepticus. *Int. J. Contemp. Pediatr.* 3:4.

[B83] Simms B. A. Zamponi G. W. (2014). Neuronal voltage-gated calcium channels: Structure, function, and dysfunction. *Neuron* 82 24–45. 24698266 10.1016/j.neuron.2014.03.016

[B84] Smets K. Duarri A. Deconinck T. Ceulemans B. Van De Warrenburg B. P. Züchner S. (2015). First de novo Kcnd3 mutation causes severe Kv4.3 channel dysfunction leading to early onset cerebellar ataxia, intellectual disability, oral apraxia and epilepsy. *BMC Med. Genet.* 16:51. 10.1186/s12881-015-0200-3 26189493 PMC4557545

[B85] Sosanya N. M. Brager D. H. Wolfe S. Niere F. Raab-Graham K. F. (2015). Rapamycin reveals an mtor-independent repression of Kv1.1 expression during epileptogenesis. *Neurobiol. Dis.* 73 96–105. 10.1016/j.nbd.2014.09.011 25270294

[B86] Sosanya N. M. Huang P. P. C. Cacheaux L. P. Chen C. J. Nguyen K. Perrone-Bizzozero N. I. (2013). Degradation of high affinity HuD targets releases Kv1.1 mRNA from miR-129 repression by mtorc1. *J. Cell Biol.* 202 53–69. 10.1083/jcb.201212089 23836929 PMC3704988

[B87] Stafstrom C. E. Carmant L. (2015). Seizures and epilepsy: An overview for neuroscientists. *Cold Spring Harb. Perspect. Med.* 5:a022426. 10.1101/cshperspect.a022426 PMC4448698 26033084

[B88] Stafstrom C. E. Staedtke V. Comi A. M. (2017). Epilepsy mechanisms in neurocutaneous disorders: Tuberous sclerosis complex, neurofibromatosis type 1, and Sturge–Weber syndrome. *Front. Neurol.* 8:87. 10.3389/fneur.2017.00087 28367137 PMC5355446

[B89] Switon K. Kotulska K. Janusz-Kaminska A. Zmorzynska J. Jaworski J. (2017). Molecular neurobiology of mTOR. *Neuroscience* 341 112–153. 27889578 10.1016/j.neuroscience.2016.11.017

[B90] Vergnol A. Traoré M. Pietri-Rouxel F. Falcone S. (2022). New insights in CaVβ subunits: Role in the regulation of gene expression and cellular homeostasis. *Front. Cell. Dev. Biol.* 10:880441. 10.3389/fcell.2022.880441 35465309 PMC9019481

[B91] Villa C. Combi R. (2016). Potassium channels and human epileptic phenotypes: An updated overview. *Front. Cell. Neurosci.* 10:81. 10.3389/fncel.2016.00081 27064559 PMC4811893

[B92] Wang H. Kunkel D. D. Martin T. M. Schwartzkroin P. A. Tempel B. L. (1993). Heteromultimeric K+ channels in terminal and juxtaparanodal regions of neurons. *Nature* 365 75–79. 10.1038/365075a0 8361541

[B93] Wang J. Lin Z.-J. Liu L. Xu H.-Q. Shi Y.-W. Yi Y.-H. (2017). Epilepsy-associated genes. *Seizure* 44, 11–20. 28007376 10.1016/j.seizure.2016.11.030

[B94] Wang H. Zhang X. Xue L. Xing J. Jouvin M. H. Putney J. W. (2016). Low-voltage-activated CaV3.1 calcium channels shape T helper cell cytokine profiles. *Immunity* 44 782–794. 10.1016/j.immuni.2016.01.015 27037192 PMC6771933

[B95] Wappl E. Koschak A. Poteser M. Sinnegger M. J. Walter D. Eberhart A. (2002). Functional consequences of P/Q-type Ca2+channel Cav2.1 missense mutations associated with episodic ataxia type 2 and progressive ataxia. *J. Biol. Chem.* 277 6960–6966. 11742003 10.1074/jbc.M110948200

[B96] Weissgerber P. Held B. Bloch W. Kaestner L. Chien K. R. Fleischmann B. K. (2006). Reduced cardiac L-type Ca2+ current in Ca(V)beta2-/- embryos impairs cardiac development and contraction with secondary defects in vascular maturation. *Circ. Res.* 99 749–757. 10.1161/01.RES.0000243978.15182.c1 16946137

[B97] Wolff M. Johannesen K. M. Hedrich U. B. S. Masnada S. Rubboli G. Gardella E. (2017). Genetic and phenotypic heterogeneity suggest therapeutic implications in SCN2A-related disorders. *Brain* 140, 1316–1336. 28379373 10.1093/brain/awx054

[B98] Wong M. (2008). Mechanisms of epileptogenesis in tuberous sclerosis complex and related malformations of cortical development with abnormal glioneuronal proliferation. *Epilepsia* 49 8–21. 10.1111/j.1528-1167.2007.01270.x 17727667 PMC3934641

[B99] Wong M. (2013). A critical review of mTOR inhibitors and epilepsy: From basic science to clinical trials. *Expert Rev. Neurother.* 13 10.1586/ern.13.48 23739003 PMC3875463

[B100] Wormuth C. Lundt A. Henseler C. Müller R. Broich K. Papazoglou A. (2016). Review: Ca(v)2.3 R-type voltage-gated Ca(2+) channels - functional implications in convulsive and non-convulsive seizure activity. *Open Neurol. J.* 10 99–126. 10.2174/1874205X01610010099 27843503 PMC5080872

[B101] Xie C. Su H. Guo T. Yan Y. Peng X. Cao R. (2014). Synaptotagmin I delays the fast inactivation of Kv1.4 channel through interaction with its N-terminus. *Mol. Brain* 7:4. 10.1186/1756-6606-7-4 24423395 PMC3896893

[B102] Yang C. Hua Y. Zhang W. Xu J. Xu L. Gao F. (2018). Variable epilepsy phenotypes associated with heterozygous mutation in the SCN9A gene: Report of two cases. *Neurol. Sci*. 39, 1113–1115. 29500686 10.1007/s10072-018-3300-y

[B103] Yu F. H. Catterall W. A. (2003). Overview of the voltage-gated sodium channel family. *Genome Biol.* 4 207–207. 12620097 10.1186/gb-2003-4-3-207 PMC153452

[B104] Yum M.-S. Lee E. H. Ko T.-S. (2013). Vigabatrin and mental retardation in tuberous sclerosis: Infantile spasms versus focal seizures. *J. Child Neurol.* 28 308–313. 10.1177/0883073812446485 22752486 PMC3695701

[B105] Zaman T. Lee K. Park C. Paydar A. Choi J. H. Cheong E. (2011). Cav2.3 channels are critical for oscillatory burst discharges in the reticular thalamus and absence epilepsy. *Neuron* 70 95–108. 10.1016/j.neuron.2011.02.042 21482359

[B106] Zamponi G. W. Striessnig J. Koschak A. Dolphin A. C. (2015). The physiology, pathology, and pharmacology of voltage-gated calcium channels and their future therapeutic potential. *Pharmacol. Rev.* 67 821–870. 26362469 10.1124/pr.114.009654 PMC4630564

[B107] Zemel B. M. Ritter D. M. Covarrubias M. Muqeem T. (2018). A-type K(V) channels in dorsal root ganglion neurons: Diversity, function, and dysfunction. *Front. Mol. Neurosci.* 11:253. 10.3389/fnmol.2018.00253 30127716 PMC6088260

[B108] Zeng L. H. Xu L. Gutmann D. H. Wong M. (2008). Rapamycin prevents epilepsy in a mouse model of tuberous sclerosis complex. *Ann. Neurol.* 63 444–453. 10.1002/ana.21331 18389497 PMC3937593

[B109] Zeng L.-H. Rensing N. R. Wong M. (2009). The mammalian target of Rapamycin signaling pathway mediates epileptogenesis in a model of temporal lobe epilepsy. *J. Neurosci.* 29 6964–6972. 10.1523/JNEUROSCI.0066-09.2009 19474323 PMC2727061

[B110] Zhang T. Chen M. Zhu A. Zhang X. Fang T. (2020). Novel mutation of SCN9A gene causing generalized epilepsy with febrile seizures plus in a Chinese family. *Neurol. Sci.* 41 1913–1917. 10.1007/s10072-020-04284-x 32062735 PMC7359139

[B111] Zhang Y. Kong W. Gao Y. Liu X. Gao K. Xie H. (2015). Gene mutation analysis in 253 chinese children with unexplained epilepsy and intellectual/developmental disabilities. *PLoS One* 10:e0141782. 10.1371/journal.pone.0141782 26544041 PMC4636363

[B112] Zou J. Zhang B. Gutmann David H. Wong M. (2017). Postnatal reduction of tuberous sclerosis complex 1 expression in astrocytes and neurons causes seizures in an age-dependent manner. *Epilepsia* 58 2053–2063. 10.1111/epi.13923 29023667 PMC5716871

